# The Effect of Real-World Personal Familiarity on the Speed of Face Information Processing

**DOI:** 10.1371/journal.pone.0001223

**Published:** 2007-11-21

**Authors:** Benjamin Balas, David Cox, Erin Conwell

**Affiliations:** 1 Department of Brain and Cognitive Sciences, Massachusetts Institute of Technology, Cambridge, Massachusetts, United States of America; 2 Department of Cognitive and Linguistic Sciences, Brown University, Providence, Rhode Island, United States of America; University of Minnesota, United States of America

## Abstract

**Background:**

Previous studies have explored the effects of familiarity on various kinds of visual face judgments, yet the role of familiarity in face processing is not fully understood. Across different face judgments and stimulus sets, the data is equivocal as to whether or not familiarity impacts recognition processes.

**Methodology/Principal Findings:**

Here, we examine the effect of real-world personal familiarity in three simple delayed-match-to-sample tasks in which subjects were required to match faces on the basis of orientation (upright v. inverted), gender and identity. We find that subjects had a significant speed advantage with familiar faces in all three tasks, with large effects for the gender and identity matching tasks.

**Conclusion/Significance:**

Our data indicates that real-world experience with a face exerts a powerful influence on face processing in tasks where identity information is irrelevant, even in tasks that could in principle be solved via low-level cues. These results underscore the importance of experience in shaping visual recognition processes.

## Introduction

The human visual system effortlessly and automatically extracts a wealth of information from face stimuli, including identity, gender, expression, race, age, and a host of other properties. For the most part, the ability to extract such information from a given face does not require extensive exposure to that particular face, and judgments of properties such as gender or race are performed with high accuracy even on completely novel faces. Even so, humans tend to encounter a relatively small number of faces repeatedly, and it is not surprising that these familiar faces may enjoy some processing advantages relative to unfamiliar faces. [1],[2],[3],[4]. However, it is not clear *a priori* that all face judgments should necessarily benefit from familiarity, nor is there any reason to believe that various kinds of judgment should benefit equally from familiarity. Variation in the advantage conferred by familiarity across tasks could provide important clues to the nature of face representations.

The conceptual orthogonality of many face judgments (e.g. the expression of a face is independent of its gender) has led to the early idea that various face recognition tasks might be executed by parallel, non-overlapping “modules” [5]. Since face familiarity ostensibly depends on the identity of a face, a strong formulation of the modular model might suggest that face familiarity should not affect other tasks, such as gender judgments, because “identity” and “gender” would be processed by separate, non-interacting modules. Along these lines, there are some results that indicate familiarity does not appear to affect gender recognition [6] or expression classification [7].

More recently, substantial evidence has emerged that familiarity does influence other “orthogonal” face judgments. Using images that were parametrically morphed along a continuum between trained (“familiar”) and untrained (“unfamiliar”) faces, Rossion demonstrated significantly faster response times (RTs) for sex classification of the familiar stimuli compared to the unfamiliar images [8]. Likewise, other researchers have pointed out cases where it appears that there are interactions between the familiarity of a face and the processing of race [9], expression [10],[11], and even speech [12]. Taken together, these studies suggest that experience with faces might exert a strong influence on tasks beyond those that are explicitly related to identity.

In the present study, we sought to further extend what is known about facial familiarity in three simple delayed-match-to-sample tasks in which subjects were required to match faces on the basis of orientation (upright or inverted), gender, or identity. We assess the extent to which familiarity with a face lessens the response time for accurate classification across these three judgments. There are several reasons why we believe this experiment fills important gaps in our understanding of familiar face processing. First, the use of a matching task minimizes the memory and training requirements necessary to carry out these three recognition tasks. Furthermore, regardless of whether the subject was matching a face according to gender, identity, or orientation, the format of the task – a binary left/right choice – was held constant across tasks. This is preferable to comparing behavior across tasks of varying formats (e.g. a binary choice such as male/female in one task and a multiple-category choice such as expression or identity in another). In addition, our use of *personally* familiar faces obviates the need for training on novel images (which may not lead to complete “familiarity”) or the use of celebrity faces (which may be more distinctive than typical faces). There is also reason to believe that personal acquaintances should give rise to the strongest familiarity effects [13]. Finally, by asking subjects to perform relatively easy matching tasks, we avoid the possibility of a speed-accuracy trade-off by looking for variations in RT while all subjects are performing highly accurately.

## Materials and Methods

### Stimuli

We used a database of faces depicting residents and affiliates of a large (roughly 150-person) undergraduate dorm at MIT. The full image set contains 190 unique individuals, half men and half women. Each individual is pictured in left and right profile, left and right ¾ view, and in two different frontal images. The pictures were initially full-color and 640×480 pixels in size.

For presentation, the images were resized to 128×96 pixels, and reduced to grayscale so that broad color cues could not facilitate recognition of targets. To make the matching tasks less trivial, target faces were also Gaussian-blurred in Adobe Photoshop to approximately 6 cycles across the face. Blurring was intended to discourage subjects from performing matching based on small-scale details like moles or blemishes on the face.

Cue images were generated for the “Gender” and “Orientation” tasks by creating facial morphs of the images in our database using MorphMan. The orientation cue image was the result of morphing together all faces in the database. For the “Gender” task, male and female cue images were created by morphing together all the men and women in the database respectively.

### Subjects

Twenty-four subjects (four men and twenty women, aged 18–25, from the MIT undergraduate community) participated in this study. Subjects were shown a collection of 190 faces and were asked to select 18 for use in the experiment. For twelve of the subjects, half of the stimuli were chosen from the subset of individuals who were highly familiar (meaning that the subject encountered these individuals multiple times per day and had known them for at least a full semester), and half of the stimuli were chosen from the subset of individuals who were not familiar (meaning that the subject could not recall having seen these individuals before). The remaining twelve subjects were gender-matched controls who had no acquaintances among the individuals in the database. Each control subject was assigned stimuli that matched the set of images selected by a subject in the experimental group. All subjects were compensated for their participation in this study.

### Procedure

Subjects were seated approximately 0.5 m from a computer monitor with no restrictions on head position. Before beginning, subjects in the experimental group were shown the entire set of individuals in the database and asked to select 9 individuals familiar to them, 5 of which were to be of their gender. They were then asked to select an additional 9 individuals (5 gender matched) who they had never seen before, or seen only infrequently (meaning once or twice). Each gender-matched control was shown the faces selected by their experimental group counterpart and asked if they recognized anyone. Volunteers for the control group who indicated that they did recognize individuals in the array were asked to participate in a different experiment not related to the present study.

**Figure 1:**
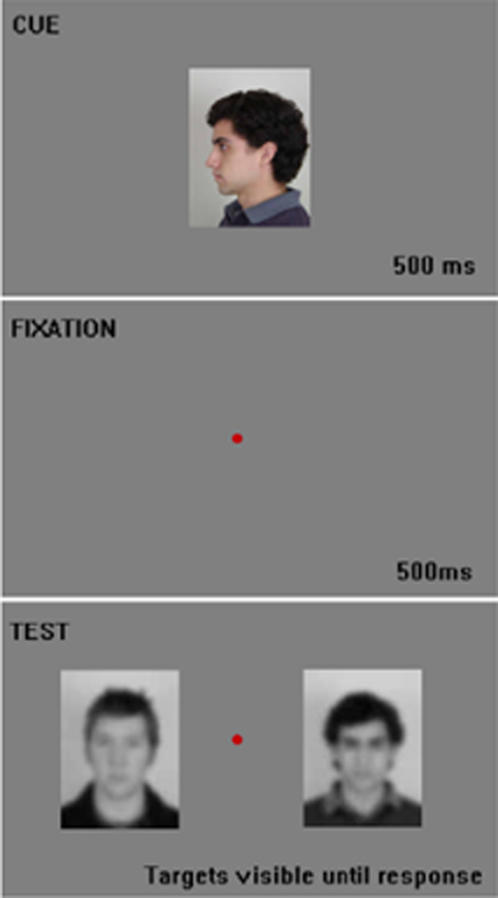
Delayed-Match-to-Sample task. An illustration of the cued 2AFC task used in all three tasks. An “Identity” trial is depicted here, with the correct answer being the right-most image.

Each subject participated in the “Orientation”, “Gender,” and “Identity” tasks, with task order balanced across subjects. In each task, a trial began with the presentation of a cue image for 500ms in the center of the screen. After a 500ms pause, the subject was then presented with two images (left and right), one that matched the cue image with regard to the current task and another that did not (Figure 1). Subjects were asked to indicate which stimulus matched the cue via button presses as quickly and accurately as possible. Target images remained on screen until the subject made a response. Location of the target was randomized across trials.

In the “Orientation” task, the cue stimulus was always the grand average morph described previously, presented upright, unblurred and in full-color. Test images were blurred, grayscale frontal images of one individual, one presented upside-down and the other presented upright. Each individual was used 4 times in this experiment, for a grand total of 36 “familiar” trials and 36 “unfamiliar” trials per subject.

In the “Gender” task, the cue image was either the average female or average male morph described previously. The cue was presented upright, unblurred and in full-color followed by blurred, grayscale test images. Test images always displayed one male and female, both drawn from the “Familiar” pool or the “Unfamiliar” pool for the subject in question. Each possible pair of differently gendered faces of the same familiarity was used twice, once with the male image as a cue, once with the female image as a cue, for a grand total of 40 trials per condition. To limit subjects' ability to utilize “pictorial information” [14] to perform the task, the particular view used for each pair of test images was rotated through the two unique frontal views and the two ¾ views available for each person.

Finally, in the “Identity” task, subjects were cued with unblurred, upright, full-color profile images of the individuals in their stimulus set. Test images were blurred, grayscale images and also matched at test for familiarity as described above. Each individual was used as a cue 4 times, for a grand total of 36 trials per condition. As in the “Gender” task, the view selected for the test images was rotated through the frontal and ¾ views for each individual.

All stimulus presentation parameters and response collection were carried out with the use of the Matlab Psychophysics Toolbox [15],[16].

## Results

If the relevant cognitive processes are truly independent of familiarity, we expect that responses to “Familiar” faces should be no faster than those to “Unfamiliar” faces. However, if facial familiarity does affect any of the recognition processes recruited to complete the three tasks described here, we should see evidence of reduced response time for correct judgments of orientation, gender, or identity matching in the experimental group. Given that the tasks we present are not difficult, we do not expect to see any variation in accuracy across subjects or tasks. To control for the fact that some faces may be easier than others to classify according to gender (or orientation and identity), we shall also directly compare the speed advantage for “Familiar” v. “Unfamiliar” faces in our experimental group to that derived from the control group. In doing so, we are able to rule out any effects of potentially confusing images that are only accurately classified if one has personal knowledge of the individual depicted.

### Accuracy

Average performance for all subjects across all three tasks exceeded 95% correct. A two-way ANOVA with subject group and task as factors yielded no significant main effects or interactions (F<1 in all cases). As we expected, all subjects found the three implementations of this matching task very easy.

### Response Time

**Figure 2:**
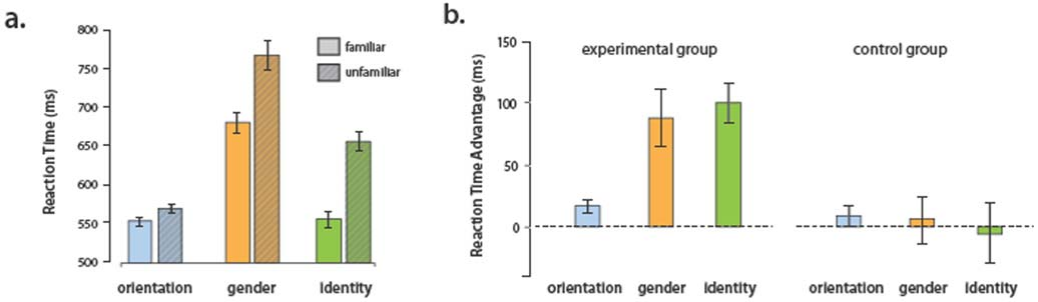
Familiarity effects on Response Time across tasks. (a) Average RT for matching across task for experimental group subjects. There is a clear advantage for familiar face matching according to gender or identity, as well as a small but significant advantage for orientation matching. (b) The mean RT advantage by task for both the Experimental and Control groups. The speed advantage conferred by familiarity for Gender and Identity matching is significantly larger in the Experimental group. The Orientation speed advantage for the Control group is not significantly greater than zero, but is also not significantly smaller than the speed advantage seen in the Experimental group. In both panels, error bars represent +/− 1 S.E.M.

**Figure 3:**
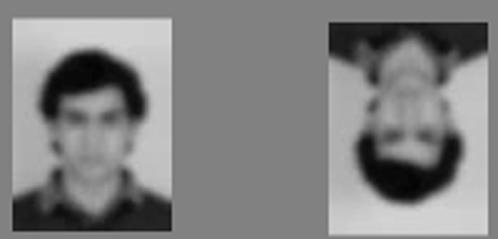
Examples of upright and inverted stimuli. Examples of upright and inverted stimuli presented to subjects in the Orientation matching task. Despite the lack of a significant two-sample difference between performance in the experimental and control groups, the profound low-level differences in these two images make it unlikely that the set of familiar faces selected by the Experimental group introduces a confounding factor in this task.

Subjects in the Experimental group were significantly faster at matching familiar faces than unfamiliar faces in all three tasks (”Identity” p<0.001, “Gender” p<0.005, “Orientation” p<0.005; planned, one-tailed t-tests; see Figure 2a). This advantage was substantial for the “Identity” and “Gender” tasks (100 ms and 88 ms, respectively), and smaller (but still significant) for the “Orientation” task (17 ms) (Figure 2b). Interestingly, for familiar faces, the “Identity” matching task could be performed as fast as the “Orientation” task, in spite of the fact that the “Orientation” task involved very large image-level differences (see Figure 3) and could, in principle, be solved using purely low-level image cues.

We continue by examining the RT data from our Control group, matched for age and gender to the Experimental group, for whom none of the faces were familiar. Each subject in this group was shown the same images shown to one subject in the original group, and their data was analyzed with the sham labels “Familiar” and “Unfamiliar,” taken from the original subjects' assessment of familiarity. These subjects showed no advantage in any of the three tasks (”Identity” p = 0.58, “Gender” p = 0.38, “Orientation” p = 0.14, one-tailed t-tests). The mean RT advantage across tasks in the Control group is displayed in Figure 2b.

Comparison of the RT advantages in the Experimental group versus the Control group yielded a robustly significant difference (of approximately the same magnitude) for the “Gender” and “Identity” tasks. The same comparison yields a non-significant difference in the “Orientation” task. It is unclear whether the lack of significance in this last comparison is due to inadequate power (arising from a small sample size, and the use of a two-sample test as compared to a one-sample test, in the original analysis), masking of the effect due to low inter-trial variability in this task compared to the others, or a genuine lack of any difference between conditions. The substantial low-level differences between upright and inverted stimuli in our task make it difficult to imagine that face familiarity was somehow confounded with ease of orientation matching (Figure 3), but we cannot completely rule out this possibility. In any event, we would stress caution in over-interpreting the observed effect of familiarity in the “Orientation” task data from our Experimental group, as it is small in magnitude.

## Discussion

We have found that real-world familiarity with a given face confers an advantage in a range of tasks, including tasks that could, in principle, be solved without processing facial identity at all. This result at least rules out the most simplistically modular models of face recognition and suggests that real-world experience with a face can exert influence over a wide range of face processing beyond the processing of facial identity.

It is not clear whether the face processing advantages seen here across tasks arise from the same basic mechanism, or from separate ones. One possibility is that early processing stages held in common between these tasks (and indeed, perhaps for all face processing) are rendered more efficient through enhanced experience, and thus all tasks are equivalently speeded. However, it is also possible that multiple, separate processes are made more efficient for faces that are familiar; the current experiments cannot distinguish between these possibilities.

A variety of mechanisms could result in speeded processing of familiar faces. One possibility is that familiarity induces a change in processing strategy. Young and colleagues [17], for instance, have previously suggested that subjects shift from using primarily external face features for recognition to relying more heavily on internal features. Such shifts in attention for often-viewed faces might better focus on information that is relevant for the tasks tested here. Techniques that can shed light on which features are useful for a given task, such as the “bubbles” paradigm [18],[19], could be particularly useful in testing this possibility. An additional possibility that is particularly relevant for our identity and gender tasks is that familiarity with a face leads to a more robust view-invariant representation of individual appearance. The influence of face familiarity on view-invariance has been discussed in some previous studies [20],[21],[22] and our results are consistent with existing data.

Another possibility is that the speed-up observed in gender processing results from the use of an alternate path to retrieve gender information. Specifically, one could imagine that identity is recognized first, and then gender is “looked up” based on stored information about this individual. Indeed, much of the speed-up enjoyed by familiar faces might result from obligatory recognition processes. If identification is automatic, familiar faces would certainly enjoy an advantage over unfamiliar faces since rapid individuation could free up resources useful for other tasks (potentially including low-level judgments like face orientation). This account requires face identification to be completed quickly relative to other processes, which is consistent with our RT data. We also note that intermixing “familiar” and “unfamiliar” faces as we have done here may have encouraged subjects to attempt to identify each presented face since there was no way to predict when a familiar individual would appear.

Finally, one might speculate that increased exposure induces the visual system to allocate relatively more representational resources to a familiar face. This idea is consistent with prototype-based accounts of face recognition [23],[24], and evidence for processing advantages for faces of one's own ethnicity (which tend to be seen more often) [25]. Even subtle shifts in representation “weight” could have a potentially large impact on face processing, as the visual system becomes better tuned to the features or configurations of features found in more frequently observed faces (e.g. more or more sharply tuned neurons tuned to features present in familiar faces). In such a scenario, more information might be available across the board for familiar faces, and this could lead to faster threshold-crossing in decisions about a variety of properties of a familiar face.

A variety of studies [26],[27],[28] have suggested that visual experience can powerfully influence visual representations and can serve as a tool to provide hints about how visual processing is organized. In that vein, the present study offers evidence that natural, real-world familiarity with a particular facial identity can influence a variety of different face tasks. Given the central importance of face processing to humans and other social primates, it is perhaps not surprising that the visual system adapts to process common facial inputs more efficiently. Further investigation of the mechanisms of such adaptation has the potential to teach us much about face processing and vision in general.
